# Ecology and evolutionary dynamics of the A-strain maize streak virus and the decade-long strategies targeting streak-resistant maize

**DOI:** 10.1016/j.virusres.2026.199810

**Published:** 2026-09-28

**Authors:** Kehinde A. Oyeniran, Temilola Aina, Lara Donaldson

**Affiliations:** a Department of Biological Sciences, Bamidele Olumilua University of Education Science and Technology, P.M.B. 250, Ikere-Ekiti, Ekiti State, Nigeria; b Plant Systems Biology, International Centre for Genetic Engineering and Biotechnology (ICGEB), Cape Town, 7925, South Africa; c Department of Integrative Biomedical Sciences, University of Cape Town, Observatory, 7925, South Africa; d Department of Molecular and Cell Biology, University of Cape Town, Rondebosch, South Africa

**Keywords:** A-strain maize streak virus, Mastreviruses, Evolution, Streak resistance, CRISPR, Gene pyramiding, MicroRNA

## Abstract

The A-strain maize streak virus (MSV-A; genus *Mastrevirus*, family *Geminiviridae*) is a single-stranded DNA (ssDNA) virus that severely constrains the production of maize in sub-Saharan Africa and other places where its insect vectors thrive. In this review, we discuss the ecology- and genomics-driven evolution of this economically important virus and strategies targeting streak resistance in maize. Evolution of MSV-A is largely driven by the interplay of mutation, recombination and selective forces that may sustain fitness and result in host adaptation. In the last decade, research efforts, including fine-mapping of quantitative trait loci (QTL), gene pyramiding, clustered regularly interspaced short palindromic repeats (CRISPR) techniques, microRNA gene silencing and farming practices, have had key roles in creating maize genotypes that are resistant to streak diseases caused by MSV-A and other plant-infecting viruses. However, these techniques may also foster the rapid emergence of editing-resistant variants, requiring more thorough and rigorous validations for desired results. We propose a five-cardinal agenda for future research covering layered surveillance-supported host resistance, evolutionary surveillance, agro-ecological alignment of breeding, escape-aware editing stewardship and policy harmonisation. Moreover, the in-depth understanding of the interplay between pathogens, hosts, and biotic interventions for breeding resistance is central to achieving crop improvement, agricultural productivity, and food security.

## Highlights

  • The A-strain maize streak virus (MSV-A), is a *Mastrevirus* in the *Geminiviridae* family.
  • MSV-A is mostly responsible for maize streak disease that constrains maize production.
  • Mutation, recombination and selection are the key drivers of MSV-A evolution.
  • Gene pyramiding and quantitative trait locus methods are useful for host resistance.
  • Molecular basis of virus evolution is central to making streak-resistant maize.

## 1 Introduction

Maize streak virus (MSV) is an important plant-infecting *Mastrevirus* in the *Geminiviridae* family (Fouad et al., 2024; Roumagnac et al., 2021). Geminiviruses represent a diverse group of economically important plant viruses that infect a wide range of susceptible plants, including both cultivated and wild species (Beam and Ascencio‐Ibáñez, 2020). The *Geminiviridae* family members are characterized by geminate particles of incomplete icosahedral symmetry, encapsulating single-stranded DNA (ssDNA) with either a bipartite (DNA-A and DNA-B) or a monopartite genome of approximately 2700 base pairs (bp) in size (Fiallo‐Olivé and Navas‐Castillo, 2023).

In sub-Saharan Africa, geminiviruses mostly constrain the cultivation and production of the most important staple food crops of grass and leguminous families (Abraham, 2024). Globally, geminiviruses are responsible for annual bean and cassava plant losses worth over two billion dollars (Beam and Ascencio‐Ibáñez, 2020). Approximately 30-90% yield reduction in cassava outputs in sub-Saharan Africa is due to African cassava mosaic virus (ACMV) with an estimated economic loss of $1-2 billion/year (Seethapathy et al., 2025). There are almost 100% tomato yield losses due to tomato yellow leaf curl virus (TYLCV) in tropical and subtropical regions (Li et al., 2022). Losses due to TYLCV and cotton leaf curl virus (CLCuV) in India and neighboring countries may exceed $500 million per year (Saurabh et al., 2021). Begomoviruses are particularly recognized worldwide as significant threats to agriculture, and over the last decade, the increasing threat posed by these viruses in various farming and cropping systems has been strongly linked to the global spread of the invasive members of their insect vector, the whitefly *Bemisia tabaci* species complex (Idrees et al., 2024).

Wild grasses are the natural reservoir of MSV, and it is believed to have emerged in maize plants from these (Harkins et al., 2009). MSV mainly negatively impacts maize production by smallholder farmers with limited to non-existent access to modern farming incentives like improved seeds, fertilizers, and pesticides. Currently, 11 MSV strains, A to K, have been described based on their genomic sequence identity, host range, geographical distribution, and degree of pathogenicity (Martin et al., 2001; Willment et al., 2002).

The A-strain maize streak virus (MSV-A) is responsible for significant economic losses in Africa, particularly in sub-Saharan Africa and other places where its insect vectors thrive and maize is the staple food crop. Subject to plant age and favorable environmental conditions, MSV-A can cause mild to severe (30-100%) yield loss in susceptible maize genotypes, and in severe outbreaks, losses may exceed $120 million annually (Tembo et al., 2020). Mounting indirect costs to farmers due to insecticide purchases for insect vector control may range between $10 and $50 per hectare, while resistant hybrids, mostly inaccessible to smallholder farmers, can be two to three times more expensive than the traditional maize varieties.

Based on the genomic sequence diversities, isolates belonging to the same species, strains and subtypes share over 75%, 91%, and 98% genomic sequence similarity, respectively (Ketsela et al., 2022). Further, the complementary and virion strands' replication-associated protein (*rep*) and coat protein (*cp*) genes are also key strategic markers for strain differentiation, considering their individual contributions to the overall genome diversity (Shepherd et al., 2010). Geographical distribution, sequence diversity, recombination, and phylogenetic evidence standards have also been leveraged for further MSV-A grouping into five subtypes that include MSV-A_1_, MSV-A_2_, MSV-A_3_, MSV-A_4_, and MSV-A_6_ (Martin et al., 2001). Owor et al. (2007) and Monjane et al. (2011) later grouped MSV-A variants into 24 recombinant lineages, of which 16 (MSV-A_1_I to MSV-A_1_XVI) are mostly MSV-A_1_ sequences with the widest dissemination rates in sub-Saharan Africa, Madagascar, the Comoros, and other islands of the Indian Ocean (Oyeniran et al., 2024).

Although MSV-A was first characterized decades ago, its genetic diversity, recombination patterns, and phylogeography have also been described in considerable detail (Fuller, 1901; Harkins et al., 2009). A broad, integrated understanding of its ecology and of how these ecological processes feed back into its evolution remains limited. Key questions still persist about how vector host preferences, environmental conditions, cropping patterns, and the distributions of both virus and vector populations have combined to shape the observed dispersal pathways of MSV-A (Monjane et al., 2011). This ecological context matters because it governs the selection pressures acting on the virus while emerging evidence of positive selection in MSV-A virion-strand genes suggests plausible host adaptation and associated virulence (Oyeniran and Tenibiaje, 2025). Without a wholistic picture of how reservoir hosts, vectors, climate, and agricultural landscapes jointly drive MSV-A evolution, it remains difficult to predict which variants will emerge, how virulence will evolve, and whether deployed resistance will remain durable. This uncertainty bears directly on the long-standing effort to breed streak-resistant maize, which remains the most effective and economically viable means of preventing maize streak disease (MSD) epidemics (Shepherd et al., 2014). Yet the durability of that effort still hangs in the balance. Essentially all MSV-A resistance reported to date may be rendered ineffective by viral genotypes capable of breaking this resistance (Shepherd et al., 2014). This is because resistance breeding has proceeded for decades against an incompletely characterized ecological and evolutionary backdrop, and a systematic assessment of these breeding strategies with the conditions under which they succeed or fail is now overdue.

This review seeks to provide a broadened understanding of the ecology and evolution of economically important MSV-A and the role it plays in virulence evolution. It also seeks to explore research insights into making streak-resistant maize in the last decades and associated challenges besetting progress in crop improvement and food security.

## 2 Ecological dimensions of MSV-A

The ecology of MSV-A is determined by the interaction of the virus with maize, wild grasses, *Cicadulina* leafhoppers, climatic conditions, and agricultural production systems. MSV-A is therefore not restricted to cultivated maize fields. It is rather maintained within a wider ecological system in which wild-grass hosts and insect vectors support virus transmission that is persistent and circulative in nature, thereby maintaining constant dispersal between cropping periods (Martin and Monjane, 2020). Wild grasses are regarded as natural reservoirs of MSV-A, and the virus is believed to have emerged in maize following adaptation from grass-associated populations (Bosque‐Pérez and Buddenhagen, 1999; Harkins et al., 2009).

### 2.1 Host reservoirs and ecological persistence

The presence of wild-grass reservoirs is important because MSV-A may persist outside the maize-growing period when susceptible maize plants are unavailable. Grass hosts can provide feeding and sheltering sites for *Cicadulina* vectors and may maintain other strains within and around agricultural landscapes. Although MSV-A causes its most severe disease in *Zea mays*, other MSV strains are more commonly associated with wild grasses, wheat, and barley (Martin et al., 2001; Willment et al., 2002). This separation is not absolute, because these strains still share ecological spaces and host communities. The coexistence of cultivated maize and wild grasses may consequently create opportunities for repeated virus acquisition, transmission, and genetic exchange.

Compared with other geminiviruses, MSV-A exhibits a relatively narrow host range; however, host range is shaped by factors beyond the virus itself. Successful infection depends on compatibility between viral proteins and host factors involved in movement, replication, systemic infection, and defense (Boulton, 2002; Martin and Monjane, 2020). These interactions link the molecular biology of MSV-A with its ecological distribution because a virus can persist in a particular environment only when suitable hosts are available.

The ecological differences between maize and wild-grass hosts may also influence MSV-A evolution. Grass-adapted strains provide a broader background of viral diversity from which maize-adapted variants may emerge. Recombination between viruses infecting related hosts can generate new genetic combinations, while selection may favor variants capable of efficient replication, transmission, or symptom development in maize (Varsani et al., 2008). Thus, the management of MSV-A requires multifocal attention to both diseased maize plants and the surrounding host vegetation supporting related viruses and vectors.

### 2.2 Vector transmission

Leafhoppers belonging to the genus *Cicadulina* are recognized as the principal insect vectors responsible for the transmission of MSV-A (Bosque‐Pérez, 2000). Among the 22 described species in this genus, at least 10 have been identified as capable of transmitting MSV-A, albeit with varying levels of efficiency (Karavina, 2014). These species include *C. arachidis, C. ghaurii, C. bipunctata* (formerly *bipunctella*), *C. niger, C. latens, C. parazeae, C. mbila, C. storeyi* (also known as *triangula*), *C. similis*, and *C. dabrowskii* (Karavina, 2014).

Relative to other *Cicadulina* species, *C. mbila* is considered the most effective vector of MSV-A due to a combination of biological, ecological, and behavioral factors. It is the most widely distributed species across diverse agroecological zones in Africa, enhancing its capacity to spread the virus over broad geographic areas (Martin and Monjane, 2020). A greater proportion of *C. mbila* individuals are competent vectors due to their female-biased populations, as females are more efficient at transmitting the virus (Shepherd et al., 2010). Following a brief acquisition period, the virus is retained in the salivary glands of *C. mbila* for prolonged periods, allowing for transmission to persist for up to five weeks (Shepherd et al., 2010).

Furthermore, seasonal morphological changes influence the species' dispersal capabilities: long-bodied forms exhibit limited movement, leading to localized infections, while short-bodied forms undertake long-distance flights, facilitating epidemic spread under drought or crop maturity conditions (Shepherd et al., 2010). Although species such as *C. storeyi* may exhibit higher individual transmission efficiency in certain regions, particularly where *C. mbila* is less prevalent, the widespread distribution of *C. mbila*, vector competence, and adaptive mobility make it the most epidemiologically significant vector of MSV-A (Martin and Monjane, 2020).

*C. mbila* specifically transmits MSV-A in a persistent, circulative, and non-propagative manner (Martin and Monjane, 2020). In this transmission mode, the leafhopper acquires the virus once during the initial feeding on an infected plant. During this process, the insect secretes saliva that solidifies into a salivary sheath around its stylets, which facilitates penetration into the plant tissue and subsequent ingestion of viral particles (Oluwafemi et al., 2007). These particles are primarily acquired from the mesophyll and parenchyma cells of the leaf, and the feeding activity often causes substantial damage to host tissues. Once inside the insect, the virus moves from the midgut into the hemolymph and eventually reaches the salivary glands (Wang and Blanc, 2021). From there, it is transmitted to healthy maize plants during subsequent feeding rounds. Fig. 1 shows the summary of the MSV-A lifecycle.

**Fig. 1:**
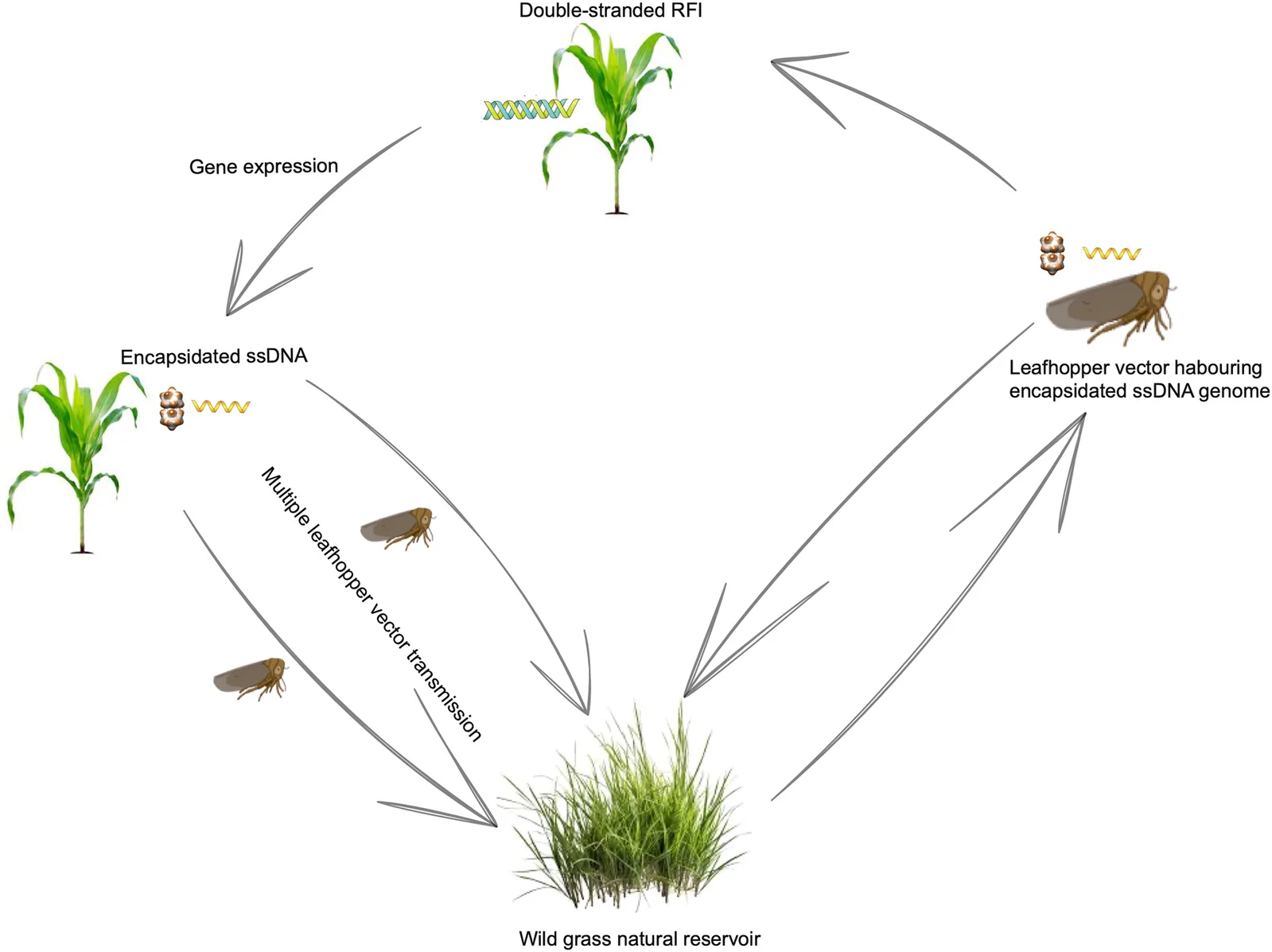
Summarised lifecycle of the A-strain maize streak virus (MSV-A). The leafhopper insect vector and the wild grass natural reservoir play a major role in the transmission and maintenance of MSV-A in and out of maize planting seasons. RFI: replicative form intermediate; ssDNA: single-stranded DNA.

Although MSV-A does not replicate within the vector, it is retained throughout the insect's lifetime following acquisition (Shepherd et al., 2010). However, only a subset of leafhoppers that acquire the virus can transmit it because transmission depends on a dominant, sex-linked genetic trait that facilitates viral movement from the gut to the salivary glands. This trait is more prevalent in female *C. mbila*, which is homozygous for the gene, and its frequency varies considerably among *Cicadulina* species (Martin and Monjane, 2020).

### 2.3 Climate and seasonal conditions

The abundance and activity of *Cicadulina* vectors are affected by environmental conditions, including temperature, rainfall, humidity, plant availability, and the nutritional status of host plants (Martin and Monjane, 2020). These factors can influence vector reproduction, development, survival, feeding activity, and dispersal. They can also affect maize growth and the availability of wild-grass hosts. As a result, the risk of MSV-A infection depends partly on the degree of synchrony between susceptible maize growth stages and periods of high vector activity.

Warm and wet conditions may also increase the availability of maize and other grasses that support leafhopper populations (Wang and Blanc, 2021). However, the relationship between climate and disease is not necessarily linear. Temperature can increase vector development within a favorable range but may also increase mortality when conditions become unsuitable (Whittaker, 2015). Rainfall may promote host-plant growth while also modifying vector movement and the duration of suitable habitats. Changes in temperature and rainfall may also alter the timing and intensity of outbreaks by affecting host growth, vector abundance, and virus transmission (Regassa, 2022). For MSV-A, climate-related changes could modify the geographical suitability of *Cicadulina* population areasand alter the period during which maize is exposed to viruliferous vectors (Benjamin et al., 2024). This possibility is particularly relevant to resistance breeding because a genotype evaluated under one vector and climatic condition may not express the same level of protection under a different ecological regime.

Furthermore, the nutritional status of host plants has a significant impact on the transmission dynamics of MSV-A. Leafhopper densities typically peak during warm and wet seasons, which coincide with the early growth stages of maize (Martin and Monjane, 2020). Consequently, planting maize during these periods of high vector activity can lead to early infections that are often associated with severe yield reductions in the field. This claim is supported by field observations from Zimbabwe and Nigeria, where *Cicadulina* populations are initially low at the onset of the rainy season but increase progressively as maize availability rises (Mylonas et al., 2014). Conversely, the decline in vector abundance during the dry season may result from the reduced availability of maize plants on which the leafhoppers forage (Mbong et al., 2021).

### 2.4 Agricultural landscapes and virus epidemiology

MSV-A transmission occurs within connected agricultural landscapes rather than isolated fields (Monjane et al., 2011). Maize fields, field margins, fallows, wild-grass habitats, and neighboring farms may all contribute to vector movements and virus-infected plant persistence. The density and distribution of host plants can influence the distance that leafhoppers travel, the frequency with which they encounter susceptible maize, and the rate at which infection spreads through a field. Host factors like the availability of suitable receptors that mediate viral attachment, entry, and replication; the efficiency of host antiviral defense mechanisms; vector specificity, such as *Cicadulina mbila*’s maize preference; and climate conditions like warmer temperatures favor MSV-A spread (Whittaker, 2015).

This landscape perspective is particularly important because *Cicadulina* populations differ in their distribution across African regions and are affected by crop density and the geographical availability of plants on which they forage (Bosque‐Pérez, 2000). Seasonal changes in crop development may consequently alter the pathways through which MSV-A moves between wild vegetation and cultivated maize (Monjane et al., 2011). The spatial relationship between reservoir hosts and maize should therefore be considered when designing disease-surveillance programs and evaluating management strategies.

MSV-A dispersal may also occur across large geographical distances. Phylogeographic analyses have identified patterns of movement and diversification across sub-Saharan Africa and the islands of the Indian Ocean (Monjane et al., 2011). The plausible anthropologically driven movement of MSV-A in and out of Madagascar further demonstrates that the virus should be examined at both local and regional scales (Oyeniran et al., 2024) (Fig. 2). In addition, the first report of MSV-A isolates from maize in the Philippines, closely related to an isolate from Zimbabwe, further emphasizes the need to investigate possible long-distance movement and anthropogenic pathways alongside natural vector-mediated dispersal (Cruz et al., 2024).

**Fig. 2:**
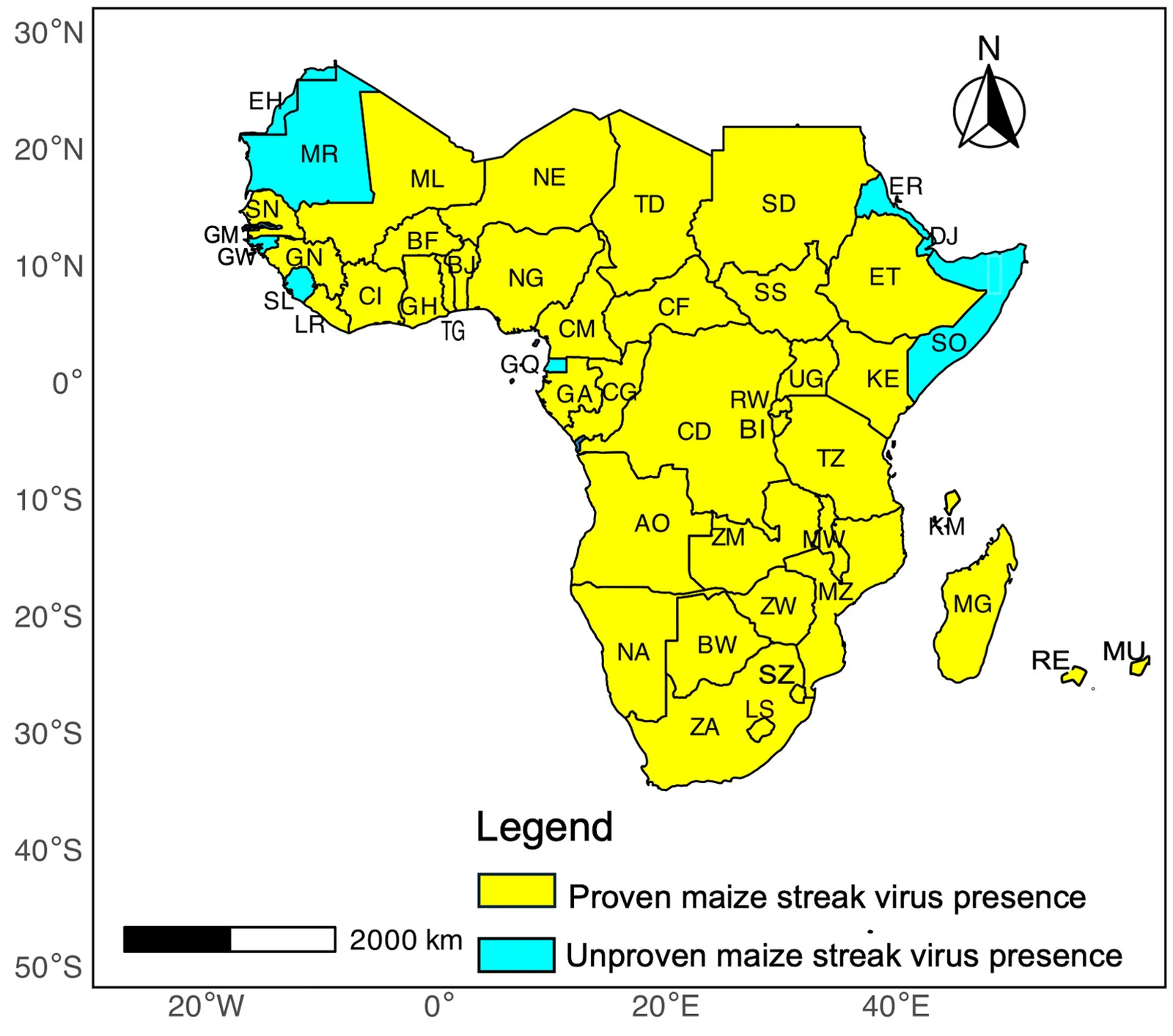
A-strain maize streak virus (MSV-A) distribution in sub-Saharan Africa and the adjacent islands of the Indian Ocean with proven endemicity and key diversification hotspots in the Eastern and Southern African regions where its insect vectors thrive (Monjane et al., 2011). Accordingly, the lack of reports from some of these countries should not be interpreted as definitive evidence of the pathogen’s absence. Infected plants usually develop symptoms between the third and the tenth post-inoculation days, while symptom severities differ by strains (Oyeniran et al., 2021; Ketsela et al., 2022; Mushayi et al., 2025). The two-letter codes for these countries are: Benin [BJ], Burkina Faso [BF], Côte d’Ivoire [CI], Ghana [GH], Guinea [GN], Western Sahara [EH], Guinea-Bissau [GW], Liberia [LR], Mali [ML], Niger [NE], Nigeria [NG], Senegal [SN], Sierra Leone [SL], Togo [TG], Angola [AO], Cameroon [CM], Central African Republic [CF], Chad [TD], Congo (Republic) [CG], Equatorial Guinea [GQ], Gabon [GA], Burundi [BI], Comoros [KM], Djibouti [DJ], Eritrea [ER], Ethiopia [ET], Kenya [KE], Lesotho [LS], Madagascar [MG], Malawi [MW], Mauritius [MU], Mozambique [MZ], Rwanda [RW], Reunion Island [RE], Somalia [SO], Sudan [SD], South Sudan [SS], Tanzania [TZ], Uganda [UG], South Africa [ZA], Botswana [BW], Eswatini (Swaziland) [SZ], Namibia [NA], Zambia [ZM], and Zimbabwe [ZW].

## 3 Virus biology/genomics

MSV-A is an ssDNA virus with a monopartite genome of approximately 2.7 kilobases with open reading frames on the viral strand containing the *mp* and *cp* genes that code for movement protein (MP) and coat protein (CP), respectively, while the complementary strand contains the *rep* gene, which, when spliced and unspliced, codes for replication-associated proteins (Rep/RepA), respectively (Fig. 3) (Shepherd et al., 2010). The MP is mainly associated with viral cell-to-cell movement and symptom development, while the CP is responsible for encapsidation of ssDNA and systemic spread (Boulton, 2002). The CP is regulated by promoters in the long intergenic region that ensure its critical roles are accomplished (Fenoll et al., 1988). While RepA regulates the host and viral gene transcription, the Rep, a spliced product of C1:C2, initiates replication and is also responsible for the activation of the virion-sense promoter for the CP (Horváth et al., 1998).

**Fig. 3:**
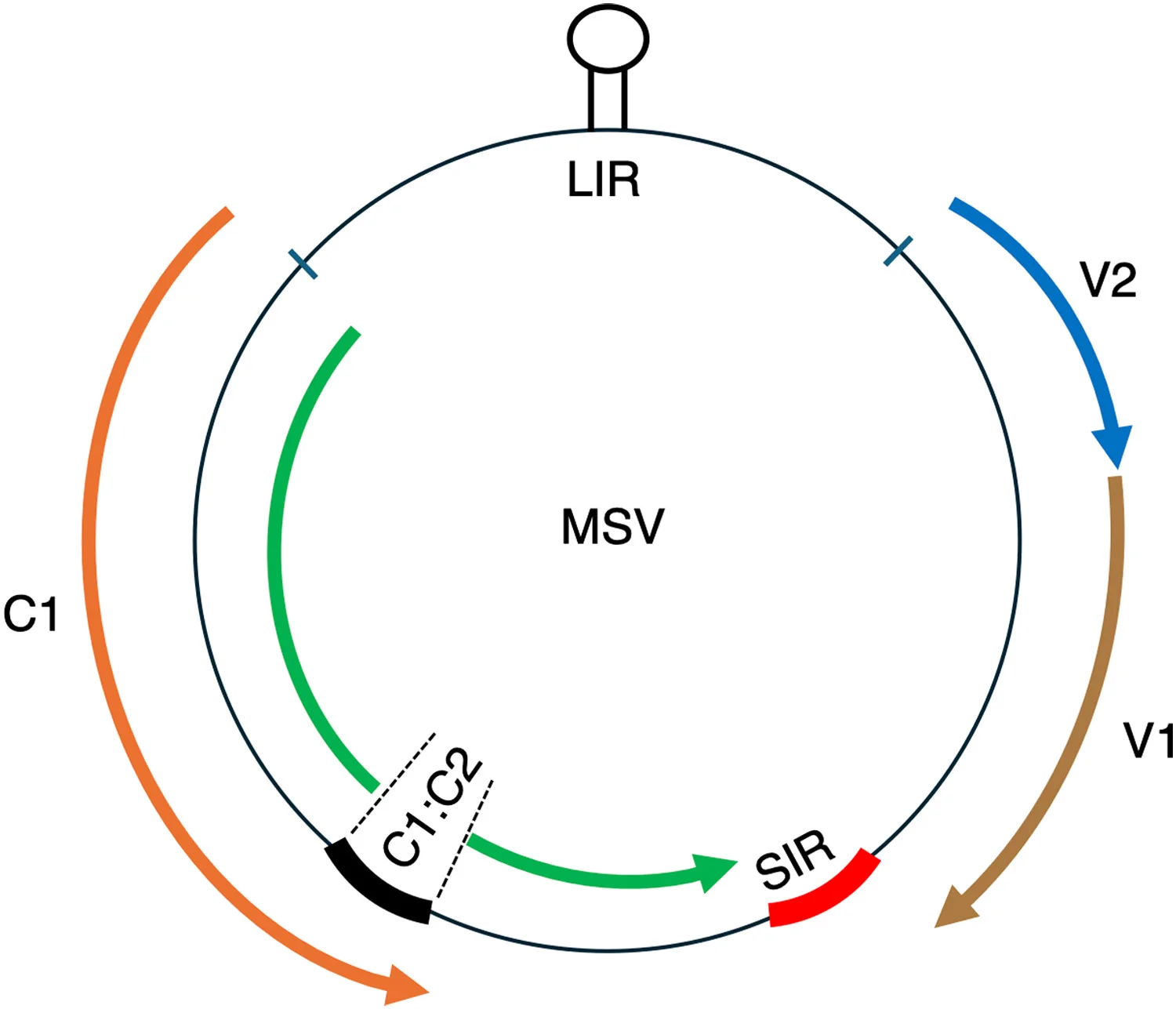
Genome organisation in A-strain maize streak virus (MSV-A). LIR: long intergenic region; SIR: short intergenic region; C1: replication-associated protein A (RepA); C1:C2: replication-associated protein (Rep); V1: coat protein (CP); V2: movement protein (MP).

A key host-specificity-determining factor in MSV-A is its genes. These genes mainly drive its evolution and adaptation to maize. The interaction of *mp* with host factors may influence which host is successfully infected (Shakir et al., 2023). The viral *mp* and the plant plasmodesmata likely have a limiting effect on the genome size, and may also indirectly affect the host range (Gilbertson et al., 2003). The *cp* controls viral DNA encapsidation and drives its transmission by insect vectors, systemic spread and symptom development in susceptible hosts. Marked variations in the *cp* may have major effects on the vector specificity and the ability of the transmitted virus to establish infection in diverse host plants (Martin and Monjane, 2020). This role directly links the *cp* with the host range, as it determines which host the virus can infect. Subject to the compatibility status of the *rep* and key host factors like the retinoblastoma protein, the *rep* must interact with the host factors successfully before replication can start (Kong et al., 2000; Muñoz‐Martín et al., 2003). Further, the relationship between viruses and plant cells in natural ecosystems may lead to host susceptibility or resistance, further stressing the importance of understanding virus-plant interactions, host range, disease symptoms, and plant defense mechanisms.

## 4 Replication

Infection starts in a susceptible host when an insect vector deposits virions into a nucleated plant cell (Martin and Monjane, 2020). The viral particles in the leaf-associated phloem tissues travel through the sieve tubes to distant parts of the plant, particularly the mesophyll sink tissues surrounding the apical meristem, a preferred site for *Mastrevirus* replication. Although actively dividing host cells like those surrounding the apical meristem are not obligatory for MSV-A replication, systemic infection and the manifestation of the characteristic streak symptoms require the virus to infect these actively dividing cells (Hanley‐Bowdoin et al., 2004).

Upon entering a suitable cell, the virion uncoats and releases the ssDNA genome into the nucleus. This process may also involve the movement of partially uncoated ssDNA into the cell nucleus by the coat protein with both DNA-binding capability and a nuclear localization signal (Guerrero et al., 2020). Inside the nucleus, the ssDNA is converted to the covalently closed circular double-stranded DNA [(ccc)dsDNA] transitional or replicative form intermediate (RFI). There are generally no alterations to host cell gene expression during this process because key cofactors and nick repair enzymes associated with negative-strand synthesis are constitutively expressed in plant cells. Following RFI transformation, the next crucial step is the expression of *rep* and *repA* genes. The Rep initiates rolling circle replication (RCR), while RepA induces the expression of cofactors and host enzymes essential for completing RCR (Hanley‐Bowdoin et al., 2004).

Interestingly, the activity of the MSV-A complementary (C)-sense promoter in maize suspension cells is observably highest during the early S-phase of the cell cycle, while the *cp* promoter shows significantly higher activity in early G2 (Nikovics et al., 2001). This timing difference correlates with the functions of MSV-A gene products. For instance, increased C-sense promoter expression during early S-phase could result in RepA-mediated manipulation of the host cell cycle via the cleavage of the retinoblastoma-related (RBR) protein. This releases regulators for transitioning from G1 to S-phase (Fig. 4). Additionally, the Rep is crucial for initiating RCR during the S-phase, while the CP is required for DNA encapsidation and systemic infection in later stages of replication (Guerrero et al., 2020).

**Fig. 4:**
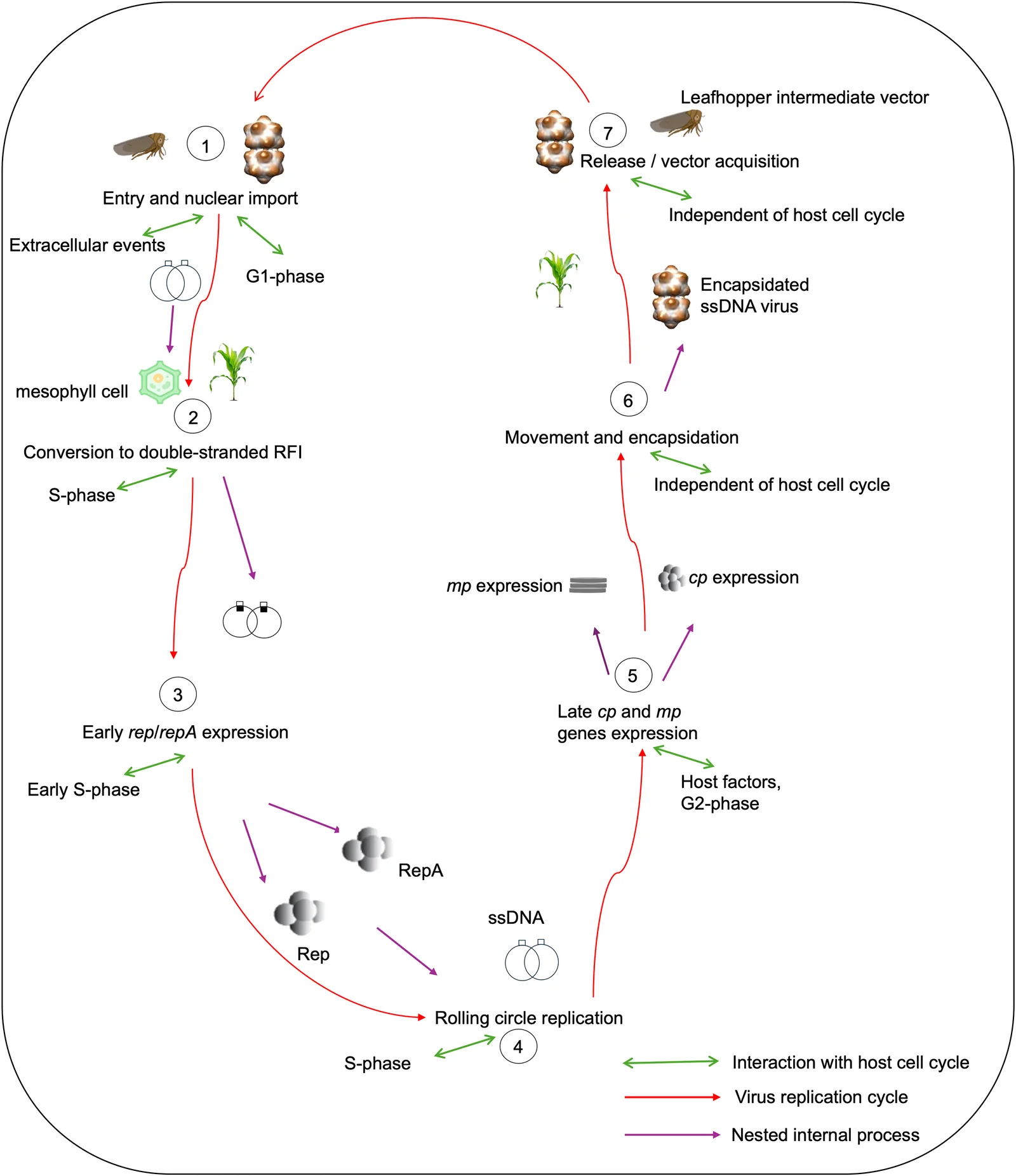
Illustration of the A-strain maize streak virus (MSV-A) replication cycle stages with gene expression activities and their interaction with the host plant cell cycle. Host-dependent processes encompass the conversion of single-stranded DNA (ssDNA) into double-stranded DNA replicative-form intermediates, expression of the replication-associated protein genes (*rep*/*repA*), rolling-circle replication, and subsequent expression of the movement (*mp*) and coat (*cp*) protein genes. Processes that are independent of the host cell cycle are entry and nuclear import, encapsidation and leafhopper vector acquisition.

Expression of the *cp* is usually time-dependent, being typically upregulated later in the infection process (Chen et al., 2020). The reactivation of the C-sense promoter in late G2 may be unrelated to *rep* or *repA* interfering with G2 phase progression, causing infected cells to remain in S-phase, preventing effective host response while maximizing virus replication (Nikovics et al., 2001). Further, the binding of Rep near the C-sense TATA box has been linked to the disruption of *rep* transcription initiation, resulting in a negative feedback process (Eagle et al., 1994).

Despite the *rep* copy number increasing exponentially during genome replication, its expression remains constant due to a tight negative feedback loop mechanism (Hovarth et al., 1998; Kong et al., 2000). The *repA* may also function as a down-regulator of viral replication, influencing various stages of infection, from preparing the cellular environment for replication to blocking the Rep later in the infection cycle and activating *cp* expression in the last stages (Bonnamy et al., 2023). The regulatory mechanisms of *repA* may occur at transcriptional, post-transcriptional (intron splicing), or post-translational phases of gene expression, covering additional processes related to RepA accumulation states (Boulton, 2002).

## 5 Evolutionary mechanisms

Evolution is the fundamental process that has shaped MSV-A into the economically important pathogen it is today. By generating and maintaining the genetic diversity that underlies the capacity of the virus to adapt to multiple hosts, overcome host defenses, and respond to changing leafhopper-grass-crop agroecosystems. This genetic heterogeneity ensures that emerging viral genomes are not identical copies of their progenitors, enabling advantageous traits to persist across successive infections and ultimately influencing the long-term fitness of viral populations.

Despite possessing a DNA genome, MSV-A evolves fairly rapidly, with a substitution rate estimated at 10^-^⁴ per site per year, which is comparable to those of RNA viruses (Oyeniran et al., 2024). This rapid evolution is further supported by the MSV-A small genome, large effective population sizes, short generation times and high mutation and recombination rates associated with its rolling-circle replication through a double-stranded replicative-form intermediate. It is also shaped by diverse selective pressures imposed by host plants, fluctuating *Cicadulina* vector populations, and changing agricultural environments (Shepherd et al., 2010; Monjane et al., 2011). In this context, evolution can be understood as any heritable changes in the genetic makeup of MSV-A over time, regardless of its duration or transience, a process that can yield viral populations with measurably improved fitness in their agro-ecological setting.

Mutation primarily drives MSV-A evolution and is the essential substrate for its adaptive change (Fig. 5). This frequent occurence of new mutations within the MSV-A populations makes the virus an exemplary model for understanding how mutation shapes viral phenotypes in plants. However, MSV-A mutation rates are only loosely correlated with its rates of evolution because most new mutations are deleterious and purged, and the high basal mutation rate does not necessarily translate one-for-one into rapid long-term genome change.

**Fig. 5:**
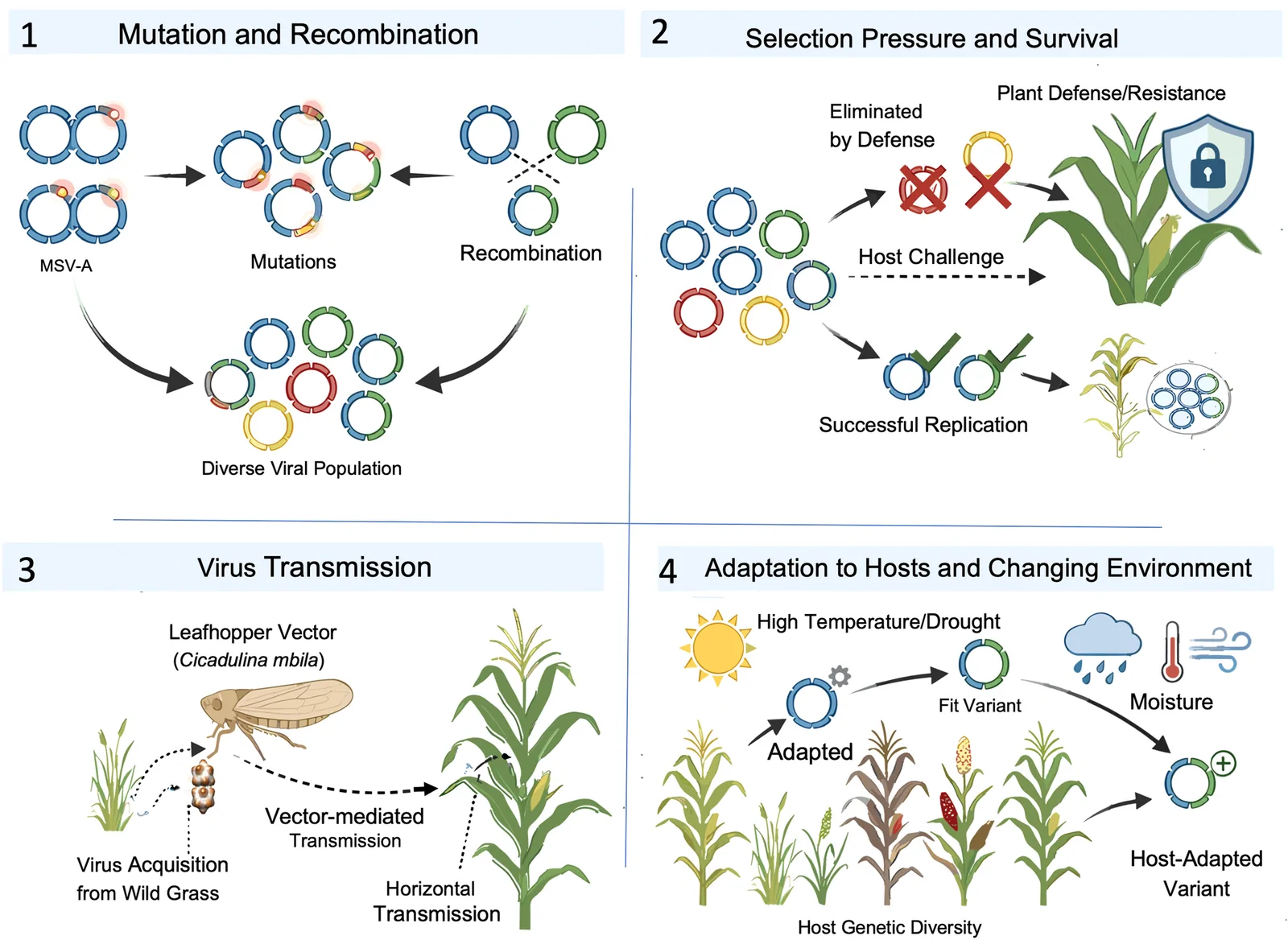
The evolutionary landscape of the A-strain maize streak virus (MSV-A) is driven by several processes and mechanisms that promote heterogeneity. The evolutionary landscape seeks to maximize the utilization of resources and ensure the survival of the most fit or adapted variants. Mutation and recombination create new genetic combinations and phenotypes. Selection pressures ensure only variants with advantageous traits survive and replicate. Insect vectors mainly aid the multi-host spread and play a role in the process of adaptation to these hosts.

Recombination is another key mechanism of virus evolution in MSV-A (Owor et al., 2007; Monjane et al., 2011). Recombination can occur between different strains of the same virus species or between different viruses by virtue of their ability to infect the same host cell. Recombination can generate genetic diversity rapidly, allowing viruses to adapt to new environments or hosts (Spielman et al., 2019). Recombination rates vary among viruses and host cells and may be influenced by factors such as the frequency of co-infection and homology. For example, recombination drives the evolution of chickpea chlorotic dwarf virus (CpCDV) (a *Mastrevirus* like MSV-A) and panicum streak virus (PanSV) with inter- and intra-species recombination, similar phylogeography and known breakpoints in *rep, cp*, and the intergenic common regions (Kanakala and Kuria, 2018; Varsani et al., 2008, 2009).

Natural selection is another critical mechanism of evolution that determines which mutants become the wild type in the population. Positive selection favors mutations that increase viral fitness, such as those that enhance replication, transmission, or immune evasion, fostering the spread of advantageous traits (Chen et al., 2021). Conversely, negative selection removes deleterious mutations that reduce virus fitness, thereby maintaining the stability of essential functions. The degree of evolution of viruses, including MSV-A, toward altered virulence is difficult to predict; however, experimental evolution in a phylogenetic framework allows scientists to study the evolution of virulence under controlled conditions, and the results support theoretical predictions like the transmission virulence-trade-off model (Berngruber et al., 2013; Monjane et al., 2020). Although the MSV-A virion strand genes seem to be mostly evolving under negative selection, evidence of detectable positive selection suggests that this too has played a role in its evolution of virulence and host adaptation (Oyeniran and Tenibiaje, 2025).

Following its emergence in maize, MSV-A appears to have evolved toward a balance between the degree of harm it causes its host and its transmission potential. This pattern is consistent with the transmission-virulence trade-off, which predicts that viruses generally evolve in ways that avoid killing their hosts before transmission can occur (Alizon et al., 2008; Monjane et al., 2020). Lastly, acquisition and transmission of MSV-A by its leafhopper vectors also have a role in the spread and evolution of the virus.

## 6 Resistance strategies

Past efforts to breed streak disease-resistant maize varieties have focused on the two main fronts that include comprehending the host's immune responses to disease agents and understanding how these pathogens infect and cause diseases in these hosts (Mushayi et al., 2025). While streak resistance research strives to leverage the understanding of the molecular basis of the host immune system for solutions, it may also benefit from major standardizations of field phenotyping protocols for viral load and symptom severity indices assessments.

### 6.1 Fine mapping quantitative trait loci/gene pyramiding

Quantitative trait locus (QTL) mapping is an effective method for discovering genomic areas linked to certain traits, such as resistance to infections by different pathogens, and genes associated with QTL in different plants, such as maize (Emeraghi et al., 2021). Numerous studies in this area, like that of Nair et al. (2015), focused on elucidating the impact of MSV-A QTL on chromosome 1. Likewise, Garcia-Oliveira et al. (2020) highlight the importance of fine-mapping the QTL on chromosome 7 for resistance to MSD in tropical maize. The frequent identification of these QTLS and meta-QTL (MQTL) with potential for broader viral disease resistance emphasises the necessity for further investigation to precisely pinpoint the genetic markers associated with resistance, aiding the development of more effective breeding strategies (Gupta et al., 2024; Mushayi et al., 2025).

In addition to MSV-A, streak disease can also be caused by non-geminiviruses (Tatineni and Hein, 2022). For instance, the molecular mechanism underlying maize resistance to streak disease caused by sugarcane mosaic virus (SCMV), a member of the *Potyviridae* family, has been identified as the *ZmTrxh* gene, an atypical h-type thioredoxin that plays a major role in resistance to SCMV in maize (Liu et al., 2017). Similar findings by Wang et al. (2022) also successfully identified *ZmLNG1* as the gene controlling various morphological traits in maize, reiterating the relevance of understanding the genetic basis of these traits for targeted improvement.

Compared to other cereal crops, maize has a limited number of qualitative resistance genes but numerous disease quantitative trait loci (dQTL) associated with variations in resistance to economically significant diseases caused by pathogens (Yang et al., 2017). Therefore, combining resistance alleles at multiple dQTL into a single line can be accomplished via phenotypic or marker-assisted selection (MAS). As such, the effectiveness of MAS depends on a comprehensive understanding of the genetic architecture governing resistance to a specific disease. The practical application of QTL discovery relies on the availability of validated diagnostic markers. Recent efforts have focused on diagnostic marker validation, primarily involving uniplex and multiplex PCR-based assays that track the *msv1* locus (Table 1). Experimental validations across different tropical maize populations confirm that these markers can accurately predict resistance phenotypes, significantly accelerating the breeding process (Tembo et al., 2020; Sime et al., 2021). Similarly, the integration of single nucleotide polymorphism markers replaced older simple sequence repeat markers, providing higher resolution for fine-mapping and ensuring that the resistance alleles are precisely introgressed without linkage drag of undesirable traits (Garcia‐Oliveira et al., 2020; Sime et al., 2021).

**Table 1:** Characteristics and activities of frequently identified quantitative trait loci.

QTL / Resistance Locus	Chromosomal Location	Phenotypic Variance Explained (%)	Source Germplasm / Mapping Population	Reference Markers	Notes	References
*Msv1*	Chromosome 1, short arm; 0.87 cM fine-mapped interval	Up to 76; range 40-76 across studies	Tzi4 (resistant) × Hi34 (susceptible) F₁-derived recombinant inbred lines (n = 87); CML202 and other resistant CIMMYT lines	bnl12.06a-npi262; gene placed 3 cM distal to npi262; candidate gene *GRMZM2G046848*	Accounts for the major proportion of resistance: source-dependent additive or partial-dominant gene action	(Welz et al., 1998; Kyetere et al., 1999; Nair et al., 2015)
18 QTLS across 11 genomic regions	Chromosomes 1, 2, 3, 4, 5, 7	3.1-21.4 per QTL (moderate to minor)	Tropical maize F₂:₃ mapping population, artificially inoculated with viruliferous *Cicadulina* (screenhouse)	948 DArT markers	Novel co-localized QTLS on chromosome 4; chromosome 7 QTL flagged for fine-mapping	(Garcia-Oliveira et al., 2020)
Minor QTL, linkage group 4	Chromosomes 3, 4	14 (early resistance); detected at LOD > 2.0	VLO73311 (resistant) × CLRCW92 (susceptible) F₂ segregating population; 100 individuals; 61 polymorphic SNPs	Tightly linked SNP markers PZA00413_20 and PZA03198_3	SNPs are applicable in MAS and can speed up breeding MSV-A-resistant maize	(Arusei et al., 2019)
umc2228, umc2229, bnlg1832	Not specified in source	93, 78.9, 71.3	MAL13 (resistant) × MAL9 (susceptible) S₄ RIL families	umc2228 (LOD 27.7); 13 polymorphic SSR markers, umc2229, bnlg1832 (LOD 18.7)	Three QTLS collectively gave 95% resistant lines.	(Lagat et al., 2008)
Additional QTLS	Chromosomes 3, 7, 9	47-51, 28-32	Multiple populations	Various SNP markers at 269 loci	Mostly germplasm- and/or environment-specific	(Ladejobi et al., 2018; Redinbaugh et al., 2018)
KASP-validated SNPs flanking *Msv1*	Chromosome 1	None reported	151 diverse early-generation inbred lines + 9 MSV-A resistant elite checks + Pool-16 (susceptible)	Three SNP markers (*Msv1*-linked) used in the KASP assay	MSV-A resistance in 151 early-generation inbreds: 133 carried the favorable alleles (54 resistant, 76 moderately resistant, 1 susceptible); screened under *Cicadulina* triangular inoculation	(Sime et al., 2021)

**QTL = Quantitative Trait Loci; CIMMYT** = International Maize and Wheat Improvement Center; **LOD** = Logarithm of the Odds; **KASP** = Kompetitive Alleles Specific PCR; **SNP** = Single Nucleotide Polymorphism; **MAS** = Marker Assisted Selection; **MSV-A =** Maize Streak Virus Strain A; **RIL** = Recombinant Inbred Lines.

Furthermore, maintaining a genome-wide heterozygous state is critical to attaining maximum heterosis in commercial hybrids, especially for dominant resistance genes, where the introgression of the resistance allele into one of the parental lines may further improve resistance performance (Mushayi et al., 2025). Other areas in which QTL fine mapping has been applied to create streak-resistant maize varieties include studies on barley yellow dwarf virus (BYDV) (chromosome 10) and maize chlorotic mottle virus (MCMV). For the latter, a key tolerance locus was identified, also on chromosome 10, within the B73 × CML333 recombinant inbred line maize population, thereby broadening our understanding of genetic factors influencing MCMV tolerance in maize (Ohlson et al., 2022).

Recent meta-QTL analysis by Gupta et al. (2024) for viral disease resistance in maize identified consensus regions across multiple viral diseases and provided a foundational framework for stacking favorable alleles to combat the global threat posed by diverse viral pathogens. Despite this progress, relatively few resistance loci have been cloned or fine-mapped to the gene level, and only a small number of resistance genes have been characterized to date. This scarcity likely reflects the polygenic complexity of many quantitative resistance traits, together with the technical challenges of validating candidate genes precisely in maize (Tao et al., 2013; Shikha et al., 2021). These setbacks are also the reason for most QTL failure.

Gene pyramiding is a beneficial approach in plant breeding that involves integrating many resistance genes into a single cultivar to enhance disease resistance. Relying on a single resistance gene (like *msv1*) for engineering is not advisable, as pathogens can evolve new virulent strains and render the gene ineffective. Empirically visualized as a pyramid-shaped structure (Fig. 6), this method systematically evaluates numerous genes in parental lines, culminating in a select few through strategic crossing experiments.

**Fig. 6:**
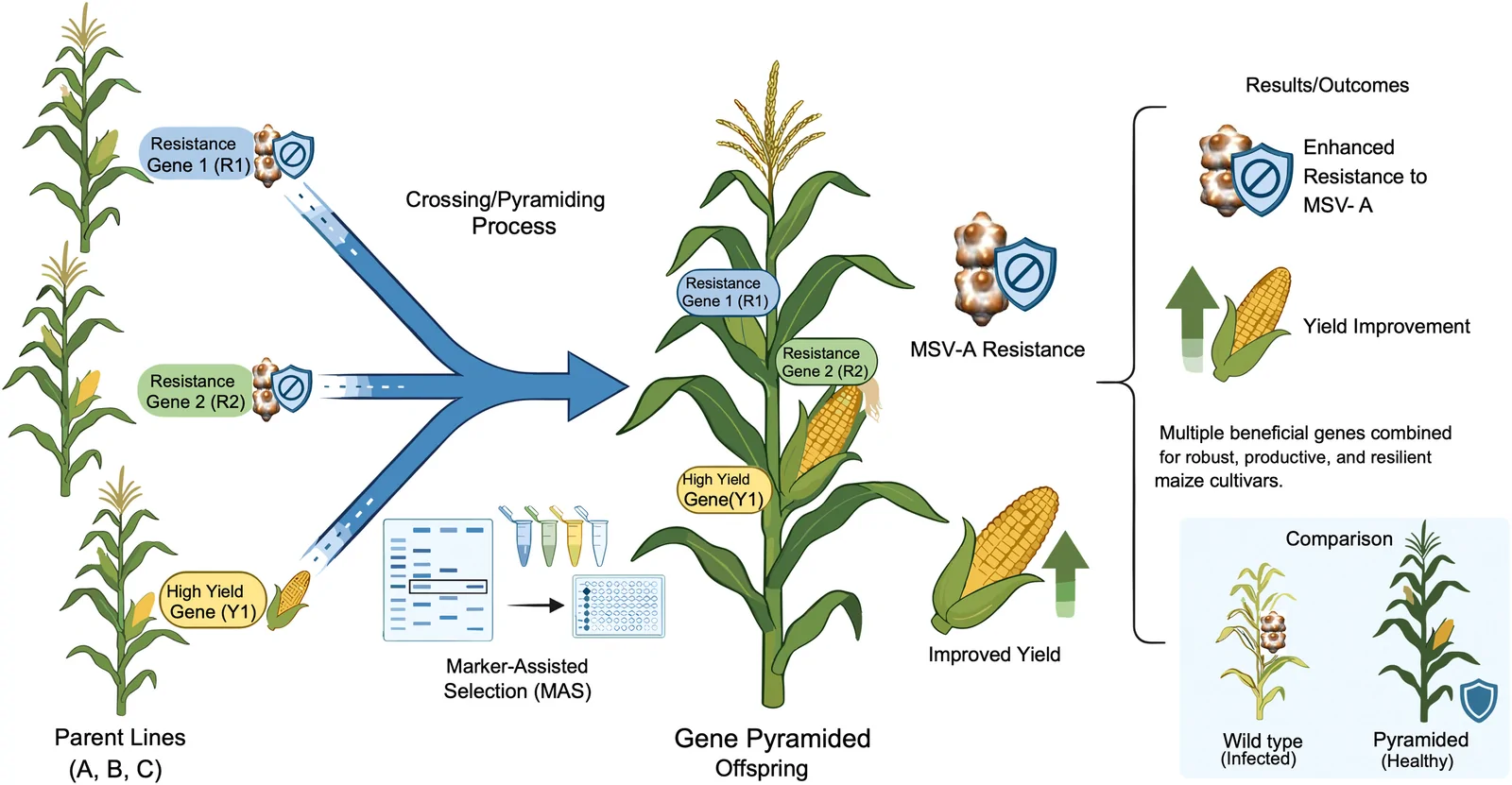
Graphical illustration summarily describes the principle, workings and end goal of the gene pyramiding technique. The illustration demonstrates how resistance to A-strain maize streak virus (MSV-A) may be strengthened by combining the major resistance genes with complementary minor-effect quantitative trait loci (QTLS) derived from diverse resistant germplasm. Marker-assisted selection (MAS) facilitates the identification of progeny carrying the desired resistance alleles, while phenotypic screening verifies their combined performance under virus challenge. The resulting resistance pyramid is expected to provide multilayered and more durable protection by reducing reliance on a single resistance locus and decreasing the risk of resistance breakdown.

Gene pyramiding can broaden the resistance base. For instance, pyramiding the major locus on chromosome 1 with minor QTLS from diverse sources (such as wild relatives or different tropical landraces) and MQTL for candidate gene mining and constitutive gene network development creates a multilayered defense that is more difficult for the virus to bypass (Zhu et al., 2021; Gupta et al., 2024; Mushayi et al., 2025). Recent breeding programs are also pyramiding MSV-A resistance with other critical traits, such as drought tolerance and resistance to other diseases (e.g., grey leaf spot or maize lethal necrosis), ensuring that new varieties are climate-smart, robust, and able to survive changing environmental conditions (Mushayi et al., 2025; Sheoran et al., 2022).

Despite these advancements, several challenges, such as the genetic background effects and associated complexity of pyramiding, are common. First, effectively deploying this strategy often requires a careful assessment of the population size to ascertain the successful recovery of genotypes harboring all targeted loci (Emeraghi et al., 2021). Second, the effectiveness of a specific QTL can vary depending on the genetic background of the maize line into which it is introgressed, thereby requiring extensive testing across different elite backgrounds (Garcia‐Oliveira et al., 2020; Zhu et al., 2021). Regarding the complexity of pyramiding, as the number of genes to be pyramided increases, the population sizes required for traditional breeding become prohibitively large (Wang et al., 2007). This is driving the adoption of computational models that predict the performance of complex pyramids before they are even crossed (Matres et al., 2021; Zhu et al., 2021).

Moreover, the durability of these stacked constructs remains a critical concern, as combining genes with distinct spectra of action may impose complex and potentially contradictory selection pressures on evolving pathogen populations (Pilet‐Nayel et al., 2017). Consequently, incorporating minor-effect QTL alongside major resistance genes can provide a more balanced evolutionary trade-off, thereby reducing the intensity of selection pressure that typically leads to rapid pathogen adaptation (Merrick et al., 2021). Lastly, combining QTLS and resistance genes (R-genes) in gene pyramiding can further aid in the attainment of long-lasting disease resistance in crops, hence guaranteeing consistent food production and minimizing economic losses resulting from infected plants.

### 6.2 Gene editing: clustered regularly interspaced short palindromic repeats

Another very popular genomic method for crop improvement is the clustered regularly interspaced short palindromic repeats (CRISPR) technique, which offers unprecedented user-friendly and cost-effective capabilities for targeted gene editing for desired traits and transgene-free plants (Gong et al., 2021). With CRISPR techniques, desired constructs carrying RNA guide(s) (gRNA) can be introduced to plants via *Rhizobium*-mediated transformation to achieve targeted mutagenesis in either the pathogen or the host (Peterson et al., 2021).

Guide RNA is a key element of the CRISPR-Cas9 gene editing system. It guides the Cas9 endonuclease to the precise target spot in the genome, enabling accurate DNA cleavage. Within the CRISPR technology, guide RNA can exist as either a natural dual RNA comprising CRISPR-RNA (crRNA) and trans-activating CRISPR-RNA (tracrRNA) or as a chimeric single guide RNA that simplifies the editing process by consolidating the essential components into a single molecule (He et al., 2024). Another key factor for a single guide RNA is the length. Research shows that guide RNAs with a length of approximately 17 nucleotides or more are often sufficient to direct Cas9 to cleave target DNA (Fu et al., 2014), while lengths of 15 nucleotides or fewer can significantly reduce editing efficiency, emphasizing the significance of gRNA length in achieving efficient genome editing. An accurate and efficient genome editing process, therefore, requires the design of effective gRNA, which is a major aspect of the CRISPR technique.

CRISPR-mediated gene editing using site-specific endonucleases has achieved success in conferring resistance to wheat dwarf virus (WDV), a *Mastrevirus* like the MSV-A (Yuan et al., 2024; Zhang et al., 2025). In a similar manner, Hu et al. (2019) developed an innovative method that utilizes a barley stripe mosaic virus (BSMV)-based guide RNA delivery system to achieve CRISPR/Cas9-mediated targeted mutagenesis in wheat and maize. Furthermore, the use of CRISPR-Cas9 ribonucleoprotein complexes for modifying the maize genome with a focus on the successful creation of gene mutations in the absence of DNA or selectable markers has also been successfully explored (Svitashev et al., 2016).

The haploid-inducer-mediated genome editing (IMGE) system for maize breeding, using the CRISPR/Cas9 technique, is also a fascinating concept that seeks to expedite the production of genetically pure inbred lines with specific traits by generating genome-edited haploids in high-quality maize varieties (Li et al., 2025; Sheng et al., 2025). Employing CRISPR/Cas9 to focus on these specific agronomic traits readily fosters the swift generation of homozygous pure lines with enhanced features, circumventing the time-consuming conventional breeding methods while effectively incorporating desirable features into high-quality commercial backgrounds.

Despite the high efficacy of CRISPR-based resistance, it also faces challenges like off-target effects, inconsistent cleavage efficiency and particularly, the rapid evolution of pathogens. To address these, it is important to carefully design guide RNAs with minimal potential for unintended targeting. This can be achieved using CRISPR design tools that analyze the target sequence in the context of the whole genome and select guide RNAs with appropriate protospacer adjacent motif (PAM) sequences. Furthermore, viral escape mutants are also common and have been documented in cassava research and other crops exhibiting imperfect cleavage by Cas9, occasionally resulting in the evolution of editing-resistant geminiviruses (Khan et al., 2022; Mehta et al., 2019). To counter this risk, researchers have leveraged multiplexed genome-editing techniques, which increase the metabolic cost of viral escape mutations and may therefore provide more durable field resistance (Abdelrahman et al., 2021; Gillani et al., 2021; Khan et al., 2022).

In addition, ethical considerations have sparked concerns across various sectors regarding the potential long-term consequences of gene-edited crops and their products on the ecosystems and the overall consumer health (Gordon et al., 2021; Ishii and Araki, 2016). Despite these challenges, gene editing techniques have nevertheless enhanced food security, a critical component of global stability and welfare.

### 6.3 RNA interference strategies

MicroRNA (miRNA) or small interfering RNA (siRNA) are short RNA molecules that do not code for proteins but have important functions in the areas of transcription regulation, biogenesis stability control and protection against drought and nutrient stress in plants (Islam et al., 2022; Zhang et al., 2022). For resistance to infections, miRNAs have been recognized in plants as major regulators of defense responses against pathogens (Kumar et al., 2022). Through the selective targeting of specific messenger RNA (mRNA), miRNAs can regulate the expression of genes that play a role in plant immunity by selectively degrading or repressing the translation of certain mRNA. (Liao et al., 2022; Yang et al., 2021). Because of this disruption, they can impact the outcome of interactions between the host plant and pathogens by upregulating the host-based defense, thereby rendering the pathogen more vulnerable to host-mediated destruction.

Over the last decade, miRNA and siRNA have emerged as promising tools for developing streak-resistant maize (Legg et al., 2019; Mushayi et al., 2025). Gene silencing using siRNA is initiated by cutting double-stranded RNA (dsRNA) into smaller pieces (miRNA and siRNA) that then bind to the RNA-induced silencing complex (RISC) (Chen, 2009). Once bound to the target gene, these small RNA molecules, via complementarity, induce repression or cleavage of the target gene by the Argonaute protein (Akbar et al., 2022; Tatineni and Hein, 2022).

The efficacy of RNAi-mediated resistance is largely determined by the design of the silencing construct (Zhang et al., 2022). More specifically, the hairpin RNA (hpRNA) is the most common approach. It involves expressing a spliceable-intron hairpin RNA that forms dsRNA, which is then processed into siRNAs by dicer-like enzymes (Mushayi et al., 2025). In maize, longer hpRNA constructs have generally proved more effective than shorter sequences for inducing silencing (Zhang et al., 2011). Artificial microRNAs (amiRNAs) have also been applied as a more precise alternative and are designed based on endogenous microRNA precursors. They offer higher specificity and reduced off-target risks and can be multiplexed to target multiple viral segments or different viruses simultaneously (Fondong, 2017; More et al., 2021; Petchthai et al., 2018). Furthermore, inducible systems have also significantly minimized the metabolic burden on the host. Researchers have developed inducible systems that ensure that transgene expression (e.g., of a mutated *rep* gene) is only triggered upon viral infection (Shepherd et al., 2014).

For instance, targeting specific genes within the 2.7 kb circular DNA genome of MSV-A is critical to effective suppression (Martin and Monjane, 2020). The *rep* gene (C1:C2) is the primary target because it is the first gene expressed upon infection and is essential for rolling-circle replication (Martin and Monjane, 2020). Rep-based hairpin constructs have demonstrated high potency in significantly reducing viral titers, while silencing the *cp* and *mp* genes targets the structural integrity and systemic spread of the virus within the plant (Owor et al., 2011). Although CP-mediated resistance is common in RNA viruses, for a DNA virus like MSV-A, targeting replication proteins often provides more robust protection (Thomson, 2007).

Previous field and laboratory RNAi trial data show that transient efficacy can be achieved in maize suspension cell assays where Rep-based hairpins demonstrated significant inhibition against MSV-A replication (Owor et al., 2011). Constructs based on specific MSV strains, including MSV-A, also demonstrated broad-spectrum potential via cross-protection against differing MSV isolates and in other mastreviruses like PanSV, albeit with a degree of resistance that correlates with sequence similarity (Shepherd et al., 2014). Stable transgenic lines expressing mutated or silenced viral genes displayed a substantial decrease in symptom severity and viral DNA accumulation, thereby indicating a significant ability to inhibit viral replication and movement (Karthikeyan et al., 2021; Shepherd et al., 2007). Moreover, gene-silencing approaches, such as spray-induced gene silencing, offer a non-genetically modified alternative, although their effects may not be durable. (Qiao et al., 2021; Rank and Koch, 2021).

However, while RNA interference can induce gene silencing, the effects are often transient due to the degradation or dilution of the interfering dsRNA intermediates within the cell and the limitation imposed by the co-evolutionary arms race between the host and the virus (Xu et al., 2019). Geminiviruses have evolved proteins, such as Rep and V2, that act as viral suppressors of RNAs to counteract the host's silencing machinery, complicating the long-term durability of RNAi-mediated resistance (Ebrahimi et al., 2023). Proteins like V2 impair the RNA-dependent RNA polymerase 6 (RDR6/SGS3) pathway via the sequestration of double-stranded RNA intermediates or the interference with the recruitment of RDR6 to its template (Luna et al., 2020). Furthermore, single-nucleotide mutations can rapidly render constructs ineffective, necessitating the development of multi-target strategies, such as stacking RNAi constructs that simultaneously silence multiple conserved viral regions to increase the genetic barrier against viral escape (Mehta, et al., 2019). Also, policy and social challenges still beset this system such that, despite technical successes, the commercialization of RNAi-based transgenic maize faces significant regulatory obstacles, public skepticism, and cultural beliefs regarding genetically modified products, particularly in the Global South (Huesing et al., 2016; Távora et al., 2022).

Overall, Tables 2 & 3 outline the strategic comparison of QTL/gene pyramiding, CRISPR-Cas genome editing, and RNA interference strategies by mechanisms, efficacy, durability, cost, regulatory requirements, stability, and regional adoptions.

**Table 2:** Comparative assessment of three resistance strategies against MSV-A.

Criterion	QTL / Gene Pyramiding	CRISPR-Cas Genome Editing	amiRNA / RNAi / HIGS / SIGS
Mechanism	Native host resistance is stacked via conventional and marker-assisted backcrossing. Leverages *Msv1* and additional mapped QTLS across chromosomes 2, 3, 4, 6, 7, 9, 10 (Garcia-Oliveira et al., 2020; Gedil and Menkir, 2019)	Direct site-specific cleavage of viral ssDNA at the sgRNA target. Nuclear expression of a transgene carrying Cas9 and one or more sgRNAs	Conversion of dsRNA to PTGS, delivered as an hpRNA transgene or as an exogenous spray (SIGS; exogenous dsRNA applied to leaves) (Shepherd et al., 2009; Rank and Koch, 2021)
Efficacy	*Msv1* explains a substantial fraction of AUDPC variance in CIMMYT-derived lines. Additional QTLS are mapped in F₂:₃ and S₄-derived germplasm, with published efficacy ranges reported across the F₂:₃ and S₄ populations (Garcia-Oliveira et al., 2020; Gedil and Menkir, 2019)	sgRNA-mediated suppression of viral replication in related geminivirus pathosystems. Site selection and multi-guide stacks enhance breadth and potency (Ali et al., 2016; Zaidi et al., 2020)	Rep-based hairpin transgenes inhibit MSV-A replication and exhibit sporadic but reliable suppression (Shepherd et al., 2014)
Durability	Highest for quantitative sources and *Msv1* stacks, which buffer against vector-pressure variability and slow resistance erosion. Major genes combined with quantitative-source pyramids substantially extend functional durability over single-gene deployments (Rex Consortium, 2016; Gedil and Menkir, 2019; Mundt, 2018)	Evasion observed in the closely related cassava-ACMV system: ∼33-48% of edited viral genomes acquired a conserved single-nucleotide insertion that abolished Cas9 cleavage (Mehta et al., 2019; Tripathi et al., 2022). Multi-guide sgRNA designs reduce but do not eliminate escape (Ali et al., 2016; Zaidi et al., 2020)	HIGS: durable when targets lie in highly conserved viral sequences, but susceptible to drift via mutagenesis in the target region (Shepherd et al., 2009). SIGS: not yet durable because spray-based delivery is operationally fragile (Das and Sherif, 2020)
Cost	Lowest entry point; front-loaded genotyping reused across cycles; molecular marker cost amortized over multiple breeding generations. Already embedded in CIMMYT-ESA and IITA pedigree pipelines (Gedil and Menkir, 2019; Masuka et al., 2016)	Highest initial cost (regeneration, containment, Cas9/gRNA stack assembly, regulatory dossier). Declines steeply once a Cas9 platform is established and shared across programs (Tripathi et al., 2022)	HIGS: comparable to the CRISPR transgenic route. SIGS: drops delivery cost significantly by avoiding stable transformation. Spray formulations are reapplied (Das and Sherif, 2020)
Regulatory	Lowest regulatory burden; conventional variety registration through NARO/NARS/CIMMYT-ESA partner systems. Cleared biosafety review in 18 SSA states with frameworks in place; commercial release has occurred (Komen et al., 2020; Prasanna et al., 2022)	Highest regulatory burden. Kenya's NBA framework and Nigeria's NBMA framework now permit confined field trials of genome-edited crops. Uganda's biosafety bill remains long-pending. Under the EU/Sweden-influenced GMO directives, foreign-DNA-free edits may be eligible for case-by-case review in some African jurisdictions (Entine et al., 2021; Komen et al., 2020; Ongu et al., 2023; Tripathi et al., 2022)	HIGS: The full GMO pathway applies in jurisdictions that follow transgenic-case regulation. SIGS: typically lower regulatory friction because no stable DNA integration is involved (Vetukari et al., 2021). Field-by-field status varies by jurisdiction and is still being clarified across SSA (Komen et al., 2020)
Stackability	Designed for stacking: *Msv1*, multiple mapped QTLS and agronomic backgrounds combined routinely in MAS-backcross pipelines	Stackable in principle (multi-guide sgRNA cassettes targeting multiple ORFs). Multi-guide reduces but does not abolish escape frequencies (Ali et al., 2016; Tripathi et al., 2022)	HIGS: stackable (multi-miRNA / hpRNA targets conserved regions). SIGS: rapid rotation between target sequences is operationally feasible because each spray batch is independent (Vetukari et al., 2021)

**QTL =** Quantitative Trait Locus (plural: loci); **CRISPR-Cas** = Clustered Regularly Interspaced Short Palindromic Repeats / CRISPR-Associated (proteins); **amiRNA =** Artificial MicroRNA**; RNAi =** RNA Interference; **HIGS =** Host-Induced Gene Silencing; **SIGS** = Spray-Induced Gene Silencing; **MSV-A =** Maize Streak Virus Strain A**; ssDNA =** Single-Stranded DNA; **sgRNA** = Single Guide RNA; **Cas9** = CRISPR-Associated Protein 9**; dsRNA** = Double-Stranded RNA**; PTGS** = Post-Transcriptional Gene Silencing; **hpRNA =** Hairpin RNA; **Rep =** Replication-Associated Protein**; ACMV =** African Cassava Mosaic Virus**; MAS =** Marker-Assisted Selection; **CIMMYT-ESA =** International Maize and Wheat Improvement Center, Eastern and Southern Africa Regional Office/Programme; **IITA =** International Institute of Tropical Agriculture; **NARO =** National Agricultural Research Organisation; **NARS =** National Agricultural Research Systems; **NBA =** National Biosafety Authority; **NBMA =** National Biosafety Management Agency**; EU =** European Union; **GMO =** Genetically Modified Organism; **SSA =** sub-Saharan Africa**; ORF =** Open Reading Frame

**Table 3:** Regional deployment status and first-line strategy for MSV-A resistance in sub-Saharan Africa.

Region	QTL / Gene Pyramiding	CRISPR-Cas Genome Editing	amiRNA / RNAi / HIGS / SIGS
West Africa	**Highest suitability.** The MAS pipeline is already compatible with regional seed systems. Documented incidence reduction under MAS-driven selection vs conventional selection in regional trial networks (Abalo et al., 2009)	**Conditional.** The NBMA 2019 amendment and draft 2020 gene-editing guidelines permit case-by-case review of SDN-1/SDN-2 edits. An MSV-A-targeting CRISPR construct carrying Cas9 + sgRNA is likely GMO-track (Komen et al., 2020; Tripathi et al., 2022)	HIGS: high regulatory friction, full GMO pathway in 18 SSA states with biosafety frameworks. SIGS: attractive because it could avoid GMO review, with stability/formulation caveats (Komen et al., 2020; Zaidi et al., 2020)
Eastern Africa	**Highest suitability.** CIMMYT-ESA marker-assisted forward breeding already drives multi-country hybrids in Kenya, Tanzania, Uganda, Zimbabwe, and Ethiopia (Prasanna et al., 2022)	**Emerging.** Kenya's NBA 2020 guidelines exempt foreign-DNA-free edits from biosafety review. Transgenic-clearance steps are still needed to reach smallholders (Akinbo et al., 2025; Entine et al., 2021; Ongu et al., 2023)	HIGS: experimental. SIGS: experimentally promising; can bypass GMO review under some African frameworks. Field validation under smallholder conditions is still pending (Das and Sherif, 2020; Endale et al., 2022)
Southern Africa	**Highest suitability.** MSV-A origin hotspot; introgressions already deployed through the CIMMYT-ESA hybrid pipeline (Lagat et al., 2008; Masuka et al., 2016; Prasanna et al., 2022)	**Limited.** No released CRISPR maize for streak virus; sequence-specific sgRNAs may mismatch newly emerged MSV-A recombinants carrying sequence variation from grass-derived relatives (Monjane et al., 2012; Varsani et al., 2008)	HIGS: pre-deployment, no commercial miRNA-MSV-A variety released. SIGS: spray-formulation stability is the binding constraint under semi-arid SSA conditions (Das and Sherif, 2020; Endale et al., 2022)
Central Africa	**Moderate suitability.** Foundational breeding capacity is developing; reported MSV-A incidence in Cameroon's agro-ecological zones I and III (abundant *Poaceae* reservoir) is higher in zones II and V. Pyramids are the first-line option until biotech products mature (Mbong et al., 2021)	**Limited.** Biotech regulatory infrastructure is still developing	**Not deployed**
Indian Ocean Island States	**First-line.** MSV-A dispersal from mainland Africa is documented. CIMMYT-ESA-derived pyramids offer an immediate intervention, while biotech products are not yet positioned for these markets (Monjane et al., 2011; Oyeniran et al., 2024)	**Limited.** Regulatory infrastructure below mainland levels	**Limited.** Regulatory infrastructure below mainland levels

**QTL** = Quantitative Trait Locus (plural: loci); **CRISPR-Cas** = Clustered Regularly Interspaced Short Palindromic Repeats / CRISPR-Associated (proteins); **amiRNA** = Artificial MicroRNA; **RNAi** = RNA Interference; **HIGS** = Host-Induced Gene Silencing; **SIGS** = Spray-Induced Gene Silencing; **MAS** = Marker-Assisted Selection; **NBMA** = National Biosafety Management Agency; **SDN-1/2** = Site-Directed Nuclease 1 (nuclease-assisted, template-free editing); **GMO** = Genetically Modified Organism; **Cas9** = CRISPR-Associated Protein 9; **sgRNA** = Single Guide RNA; **SSA** = Sub-Saharan Africa; **CIMMYT-ESA** = International Maize and Wheat Improvement Center, Eastern and Southern Africa Regional Programme; **NBA** = National Biosafety Authority; **MSV-A** = Maize Streak Virus, A-strain; **miRNA** = MicroRNA.

### 6.4 Farming practices

Despite being less effective than host plant resistance, integrated disease management strategies, involving various farming practices aimed at conferring resistance to MSV-A, have also been employed in the last decade (Mushayi et al., 2025). However, care must be taken to ensure these practices do not exacerbate disease outbreaks. For instance, while certain agricultural methods may suppress insect vectors, their indiscriminate application could lead to the development of pesticide resistance, thereby undermining the long-term efficacy of such interventions.

Improvements in pest control, rogueing, and crop rotation may also hold great promise for overall better crop management. For example, using environmentally friendly or bio-based pesticides to control the MSV-A insect vector may help lessen the long-term effects of chemical pesticides on the environment. Crop rotation is a key agricultural practice that can disrupt the life cycle of the leafhopper vectors and reduce inoculum levels (Garcia-Oliveira et al., 2020; Mushayi et al., 2025). Nevertheless, its implementation is fraught with several challenges, like the limited land availability for smallholder farmers and the economic pressure to continuously cultivate maize, which is often their primary cash crop. While crop rotation is a valid concept, its efficacy against *Cicadulina* leafhoppers is limited because these insects are highly mobile and have a broad range of alternate hosts in many wild grasses.

Rogueing is a central field-hygiene measure for managing MSD that involves the physical removal and destruction of infected plants (Karavina, 2014). In African maize agro-ecology, infected crops should be rogued and buried as soon as they are identified during the growing season, because every unrecovered infected plant acts as a source of inoculum that leafhoppers can acquire and amplify across neighboring farms. Rogueing is most effective during the early stages of an outbreak, when inoculum pressure and vector populations are still building and coordinated rogueing across contiguous fields can interrupt local spread (Jeger et al., 2023; Karavina, 2014).

However, the feasibility of repeated rogueing under smallholder conditions is constrained by labor availability, the inconvenience of destroying plants that may still yield food, and the persistent presence of grassy and cereal reservoirs around field margins that reintroduce inoculum after rogueing ceases (Mushayi et al., 2025). Rogueing should be regarded as a complementary measure rather than a replacement for resistant cultivars, rotation with broad-leaved crops, and vector management. Its intensity and timing should be tailored to local epidemiological conditions, including vector density, the proportion of infected plants, and the seasonal progression of MSD.

## 7 Future perspectives

The future management of MSV-A will depend less on a single technological breakthrough than on the capacity to conceptualize it as a dynamic ecological and evolutionary system rather than a static agronomic problem. The MSD challenge reflects the virus’s ability to cross agro-ecological boundaries, recombine with grass-adapted variants, and adapt to changing selection pressures, including those imposed by emerging biotechnological interventions (Monjane et al., 2012; Shepherd et al., 2009; Varsani et al., 2008). We propose a five-cardinal approach for future management strategies as follows:

i. Layered, surveillance-supported host resistance

Layered host-resistance and surveillance programs should combine major-gene resistance with quantitative sources and use marker-assisted backcrossing within conventional pedigree-breeding schemes. Where appropriate stewardship and regulatory conditions permit, genome-editing strategies could target conserved intergenic regions associated with multiple open reading frames, thereby increasing redundancy in resistance designs.

ii. Evolutionary surveillance

Evolutionary dynamics should inform decision-making through routine surveillance during biotechnology deployment. Phylogeographic analyses should complement field epidemiology to facilitate the early detection of emerging lineages. Surveillance should extend beyond maize to grass and cereal reservoirs harboring MSV-B through K strains, which may recombine with MSV-A in maize (Martin et al., 2001; Varsani et al., 2008, 2009; Willment et al., 2002).

iii. Agro-ecological alignment of breeding

Breeding programs should specifically integrate MSV-A screening into multi-site, multi-vector evaluation networks rather than relying on single-station trials. They should also rotate major and quantitative resistance sources across years and environments to reduce selection pressure and slow viral adaptation.

iv. Escape-aware editing stewardship

Future CRISPR and genome-editing approaches should explore recent advances in artificial intelligence and machine learning methods for food security to design and evaluate multiplexed single-guide RNAs targeting Rep-binding iterons, *mp, cp*, and the conserved nonanucleotide sequence (Bonnamy et al., 2023; Oyeniran and Donaldson, 2026). Escape-frequency benchmarks should be established using evidence from analogous geminiviral systems. Because viral escape may occur rapidly when interventions directly target and mutagenize the viral genome, effective MSV-A editing strategies will require rotating target sites across years, combining editing with conventional resistance genes, and continually releasing improved varieties.

v. Policy harmonization

Policy-guided biosafety frameworks vary substantially across sub-Saharan Africa, with direct implications for international research and development collaborations involving transgenic and gene-edited maize. The future MSV-A agenda should therefore promote review procedures that accommodate rapid editing-stack redesign, surveillance-based retirement of resistance sources once escape frequencies exceed predefined thresholds, and transparent protocols for transgene clearance.

## 8 Summary of current knowledge and paper contribution

Decades of MSV-A research have produced a robust framework spanning molecular evolution, vector biology, agroecology and resistance breeding (Bosque‐Pérez, 2000). Phylogenetic analyses have resolved at least 11 strains (A-K), grouped MSV-A into five subtypes (A_1_-A_6_) and 24 recombinant lineages, and identified eastern and southern Africa as the principal diversification hotspots from which the virus continues to spread (Monjane et al., 2011; Oyeniran et al., 2024). The ecological link between virus persistence and crop loss is now well characterized. Wild-grass reservoirs, persistent circulative transmission by *Cicadulina* leafhoppers, most notably *C. mbila*, and climate-driven shifts in vector abundance generate the erratic 3-10-year epidemic cycle that underpins the US$120-US$480 million average annual maize losses in sub-Saharan Africa (Tatineni and Hein, 2022; Tembo et al., 2020). Resistance breeding remains anchored by the major QTL *Msv1* on chromosome 1 (explaining up to 76% of the area under the disease progress curve [AUDPC] variance), augmented by minor-effect QTLs mapped on chromosomes 2, 3, 4, 6, 7, 9 and 10, and by kompetitive allele-specific PCR (KASP)-validated markers that are now implemented in the International Maize and Wheat Improvement Center (CIMMYT) forward-breeding pipelines (Garcia‐Oliveira et al., 2020; Gedil and Menkir, 2019; Sime et al., 2021). The emergence of CRISPR/Cas and miRNA tools also broadened the technical landscape, yet escape frequencies of 33-48% documented in analogous ACMV cassava systems and the limited durability of single-target designs show that no single intervention can substitute for layered, surveillance-supported resistance (Rex Consortium, 2016; Mehta et al., 2019; Mundt, 2018).

It is against this backdrop that this review makes three contributions beyond existing findings. First, it consolidates recent developments and advances in MSV-A ecology and epidemiology, movement, selection-driven evolution, and synergistic interactions with co-infecting viruses into a single ecological-evolutionary framework that explicitly links its molecular evolution to the agro-ecological conditions selecting for it (Abraham, 2024; Cruz et al., 2024; Oyeniran et al., 2024; Oyeniran and Tenibiaje, 2025).

Second, rather than cataloguing each technology in isolation, it quantitatively benchmarks three intervention classes on efficacy, durability, regulatory burden and field applicability across sub-Saharan African contexts, foregrounding the operational implications of viral escape for the next decade of breeding pipelines (Ali et al., 2016; Endale et al., 2022; Fondong, 2017; Garcia‐Oliveira et al., 2020; Mehta et al., 2019).

Third, it proposes a five-cardinal agenda: layered resistance, evolutionary surveillance, agro-ecological alignment of breeding, escape-aware editing stewardship, and policy harmonization. These serve as a coordination template for advancing MSV-A management from a static breeding objective to a dynamic, surveillance-coupled control system (Rex Consortium, 2016; Jeger et al., 2023; Mundt, 2018; Ongu et al., 2023; Tripathi et al., 2022). Taken together, this manuscript positions itself as an operational roadmap for translating accumulated ecological and evolutionary insight into durable streak resistance in the maize farming systems most vulnerable to MSD epidemics.

## 9 Conclusion

The evolutionary arms race between pathogens and their hosts is an ongoing process driven by innate survival instincts. In MSV-A, this dynamic interplay is shaped by selection, mutation, and recombination. Despite successes in the last decade, strategies for developing streak-resistant maize are, however, not fully leveraged for MSV-A, necessitating a need for more innovative approaches that readily consider the molecular underpinnings of its evolution. Most importantly, the future of MSV-A management depends on the willingness to leverage viral ecological and evolutionary dynamics, recognising that surveillance and breeding are inseparable, that resistance must be layered rather than singular, and that ecological-evolutionary surveillance, biotechnological innovation, and enabling policy frameworks must advance in parallel if MSD is to be reduced from a chronic to a marginal constraint on African maize farming.

## Funding

The authors are grateful to the International Centre for Genetic Engineering and Biotechnology (ICGEB) for sponsorship.

## Declaration of generative AI and AI-assisted technologies in the manuscript preparation process

The author(s) used the services of AI for scientific illustrations to make Fig. 5, Fig. 6, and the authors have made reasonable efforts to ensure accuracy, including additional editing and review by the authors wherever possible.

## CRediT authorship contribution statement

**Kehinde A. Oyeniran:** Writing – review & editing, Writing – original draft, Software, Methodology, Investigation, Formal analysis, Conceptualization. **Temilola Aina:** Formal analysis, Writing – original draft. **Lara Donaldson:** Writing – review & editing, Validation, Supervision, Project administration, Funding acquisition, Conceptualization.

## Declaration of competing interest

None declared.

## Data Availability

No data was used for the research described in the article.
